# Low-cost, open-access sensorized aerial robot multirotor testing operational platform

**DOI:** 10.1016/j.ohx.2025.e00732

**Published:** 2025-12-13

**Authors:** Mario Aguilera-Ruiz, Alejandro Galaviz-Mosqueda, Benjamín Jaramillo-Ávila, Salvador Villarreal-Reyes

**Affiliations:** a Monterrey Unit, Centro de Investigacion Cientifica y de Educacion Superior de Ensenada, Apodaca, N.L., C.P. 66629, Mexico; b Centro de Investigacion Cientifica y de Educacion Superior de Ensenada, Ensenada, B.C., C.P. 22860, Mexico

**Keywords:** *Aerial robot*, Open-source, Multi-rotor

## Abstract

Mathematical modeling and simulation of aerial robotic systems (ARS) constitute a highly relevant field in domains such as smart cities and Industry 4.0. In this context, validating algorithm performance under real-world conditions remains essential. However, real-world testing presents several challenges, including the isolation of scenario-specific effects on algorithm performance.

In this paper, we introduce the Low-Cost, Open-Access Sensorized Aerial Robot Multirotor Testing Operational Platform (ARMTOP). The ARMTOP features a gyroscope mounted on a fixed frame, allowing precise testing of pitch, yaw, and roll angles. Additionally, it integrates a Wi-Fi-based communication module for both sending commands and receiving onboard IMU data. The platform also includes a Graphical User Interface (GUI) for real-time visualization of IMU data, with the capability to export received data for offline analysis (e.g., feature extraction). The components of ARMTOP are developed using open-access frameworks, enhancing the platform’s replicability for further customization and development.

## Graphical abstract

**Figure:**
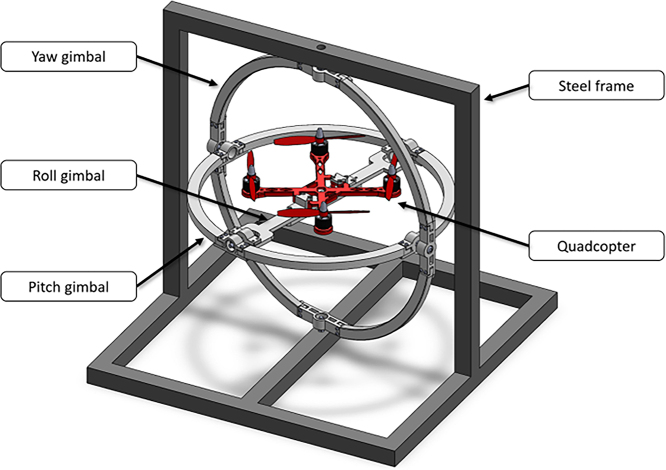

**Specifications table**

**Table:** 

Hardware name	Low-Cost, Open-Access Sensorized Aerial Robot Multirotor Testing Operational Platform
Subject area	•*Engineering and material science* •*Educational tools and open source alternatives to existing infrastructure*

Hardware type	•*Electrical engineering and computer science*

Closest commercial analog	FFT Gyro [1]

Open source license	GNU GPL 3.0

Cost of hardware	∼186$ US

Source file repository	https://doi.org/10.17605/OSF.IO/DG9PE

## 1 Hardware in context

Research and development of aerial robotic systems (ARS) represent a highly relevant area in the domains such as smart cities and Industry 4.0 [2], [3]; surveying infrastructure; facilities, or regions [4] and monitoring natural environments and agricultural areas [5]. The development of devices aimed at addressing problems such as pollution monitoring is pivotal to scalable solutions in smart cities. This includes problems such as monitoring air pollution, advancing understanding of pollutant behaviors, and supporting environmental management [6]. The development and optimization of control algorithms are essential for ARS [7], especially to enable coordinated motion among interacting swarm members while ensuring robustness and fault tolerance.

In this context, mathematical modeling and simulation serve as indispensable and extensively utilized tools. However, validating algorithm performance under real-world conditions remains essential to confirm the results observed during simulation. Real-world testing poses several challenges, with one of the most significant being the isolation of scenario-specific effects on algorithm performance [8]. For example, in the case of aerial robots, it is crucial to distinguish the effects of environmental factors such as wind, sensor noise, and mechanical errors from those caused by suboptimal parameter selection [9]. Consequently, testing methodologies that allow for the exploration of these effects under controlled conditions are essential. This is particularly relevant in scenarios where novel control mechanisms are being developed [10], [11] or where the energy consumption of an ARS is being evaluated [12], as a means to complement energy-efficient communication protocols [13] and to facilitate the deployment of energy-efficient networks. Such considerations are especially significant for applications in precision agriculture.

In this paper, we introduce a Low-Cost, Open-Access Sensorized Aerial Robot Multirotor Testing Operational Platform (ARMTOP). The ARMTOP’s building blocks are illustrated in Fig. 2. As depicted, the ARMTOP includes a gyroscope mounted on a fixed frame, enabling precise testing of pitch, yaw, and roll angles. Furthermore, it incorporates a communication module that facilitates the transmission of commands and the retrieval of data from the onboard Inertial Measurement Unit (IMU) via Wi-Fi. At the receiving end, the platform provides a Graphical User Interface (GUI) for real-time visualization of IMU data. Additionally, the received data can be exported as a CSV file for offline analysis, enabling the extraction of features in both time and frequency domains.

A comparable proprietary platform, FFT-Gyro [1], integrates a gyroscope-based sensing system with an embedded graphical user interface (GUI) and a communication module. While FFT-Gyro represents a mature and robust commercial solution, the ARMTOP platform differentiates itself primarily through its replicability and customization accessibility. FFT-Gyro is manufactured using aluminum and carbon fiber components, requiring CNC machining and composite manufacturing techniques such as tooling and mold fabrication. Consequently, reproducing or modifying FFT-Gyro demands specialized equipment and expertise, making it less accessible for laboratories or developers without advanced fabrication capabilities. In contrast, ARMTOP has been designed with accessibility and low-cost replication in mind. Most of its structural components are optimized for fabrication with standard desktop 3D printers, and the complete set of printing files and assembly documentation is freely available online. This design philosophy allows ARMTOP to be replicated with minimal resources, making it especially suitable for early-stage research, prototyping, and educational environments where adaptability and iterative development are essential. It is important to note that this distinction should not be interpreted as an inherent advantage or disadvantage of either system. Instead, ARMTOP is a required complement to platform such as FFT-Gyro. The former serves as a flexible and easily customizable platform ideal for initial development and experimentation, whereas FFT-Gyro can be employed as a fine-tuning or validation tool in advanced stages of product or system optimization. Furthermore, the communication and software frameworks of ARMTOP are entirely open-source, enabling seamless integration with analytical and data-processing tools. The inclusion of wireless connectivity, rather than a fixed USB interface, further enhances its portability and versatility, facilitating not only research applications but also its use in classroom demonstrations and public science outreach.

The proposed ARMTOP platform contributes to the field by providing a reproducible and open-access testbed for evaluating aerial robotic systems under controlled conditions. A key advantage is the explicit characterization of the platform’s moments of inertia, which enables the decoupling of the testbed’s influence from that of the ARS controller. This allows researchers and educators to obtain more accurate insights into controller dynamics, stability, and response times. Beyond immediate applications, ARMTOP also offers a base for extending experimental studies toward more complex scenarios, including robotic-assisted procedures. For instance, ARMTOP can be used to experimentally test parameters — such as controller response time — that may later be incorporated into simulation and modeling studies to better represent real-world conditions in scenarios such as construction 3D printing performing printing and evaluation tasks [14].

This paper is organized as follows. Section 1 presents the hardware in context, outlining related solutions and highlighting the motivation for the present design. Section 2 presents the detailed hardware description. Section 3 summarizes the design files and provides access to the open-source documentation. Section 4 details the bill of materials, including key components and associated costs. Section 5 presents the build instructions, describing the steps required to replicate the hardware. Section 6 provides the operation instructions, focusing on safe and effective use. Finally, Section 7 reports the validation and characterization of the hardware, demonstrating its performance and limitations in representative applications.

## 2 Hardware description

The proposed ARMTOP platform is composed of four main modules that work together to enable controlled and reproducible testing of multi-rotor flight dynamics. The mechanical foundation of the system is the **MrotorDock (MRD)**, a fixed steel frame designed to hold a set of three gimbals or pivoted supports, mounted concentrically with orthogonal pivot axes. This configuration allows a small multi-rotor drone mounted at the inner support to rotate freely along roll, pitch and yaw angles. The components of the MRD are divided into smaller parts that can be fabricated using standard 3D printers with a 200 × 200 mm print bed. Once assembled, the structure can accommodate quadcopters with a diagonal rotor distance of up to 200 mm and propeller sizes of up to 127 mm. This modular and printable design significantly reduces manufacturing costs and facilitates rapid replication in academic or research settings. Mounted within the MRD is the **QuadTestModule (QTM)**, a 3D printed quadcopter frame tailored to the mechanical limits of the testbed. The QTM integrates four 2480 KV brushless motors and corresponding electronic speed controllers managed by a Skyline32 flight controller running the Clenaflight open-source firmware [15]. This configuration provides a transparent control environment, enabling users to adjust and replicate flight parameters easily. The detailed Bill of Materials (BOM) for this module is provided in 4. Incorporating all the necessary components and instructions to build a QTM ensures swift platform validation and provides an accessible entry point for new users exploring the field.

**Fig. 1:**
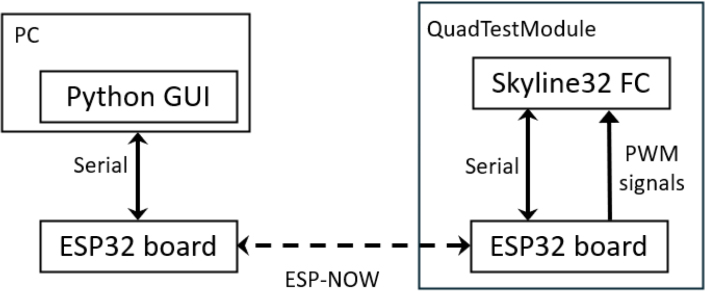
Diagram of the communication flow between the GUI and the Flight Controller in the quadcopter.

Communication and control between the QTM and an external computing system are handled by the **Telemetry and Command Module (TCM)**. This subsystem based on the ESP32 development board can be used to perform on-line fine-tuning of the PID controller gains, providing data for visual feedback (attitude angles) and a quantitative feedback (off-line analysis). TCM reduces complexity by allowing real-time communication and removing the need for a separate, dedicated radio control transmitter and receiver. This can save time, reduce setup requirements, and improve ease of use for operators. This module uses the ESP-NOW wireless communication protocol for direct and quick response [16]. Two ESP32 boards are used for this TCM. One board is connected to a PC, while the second is connected to the quadcopter’s flight controller. The onboard module connects to the flight controller using serial communication and the MSP protocol [17] to retrieve sensor data and adjust PID controller parameters. To inject control commands into the flight controller, the ESP32 board generates PWM signals that emulate those produced by a standard RC transmitter/receiver. The board connected to the PC communicates via USB serial connection, enabling data exchange with the Python-based GUI. Fig. 1 shows a diagram of the communication flow between the GUI and the QuadTestModule.

At the operator’s end, a **Python-based Graphical User Interface (GUI)** provides real-time visualization of attitude data, PID tuning controls, and the ability to log data for offline analysis. The GUI’s open-source implementation ensures cross-platform compatibility and allows seamless integration of additional analytical tools. Through this interface, the user can issue flight commands, monitor the drone’s dynamic response, and record time-stamped data for quantitative evaluation.

Together, these four modules form a cohesive system that allows the precise control, monitoring, and analysis of multirotor behavior under reproducible laboratory conditions. The integration of open-source hardware and software elements ensures transparency, adaptability, and accessibility, making the ARMTOP platform a valuable tool for educational and research applications in aerial robotics.

In summary, the proposed device can support research on multirotor aerial robots by:

• Enabling controlled and repeatable testing of multirotor dynamics, without the risk and variability of free flight experiments.
• Providing a safe and low cost environment for validating flight controllers, tuning strategies and stability margins.
• Supporting educational demonstrations and training activities, giving students a safe platform to understand key concepts in aerial robotics, such as attitude estimation and control strategies.

## 3 Design files summary

**Table:** 

Design filename	File type	Open source license	Location of the file
outer_arc	STL file	GNU GPL 3.0	https://osf.io/wyfth
outer_coupling	STL file	GNU GPL 3.0	https://osf.io/rh5nu
middle_arc	STL file	GNU GPL 3.0	https://osf.io/jaxgm
middle_coupling1	STL file	GNU GPL 3.0	https://osf.io/th4wq
middle_coupling2	STL file	GNU GPL 3.0	https://osf.io/wfh5x
inner_support1	STL file	GNU GPL 3.0	https://osf.io/2y73w
inner_support2	STL file	GNU GPL 3.0	https://osf.io/qbytg
inner_coupling	STL file	GNU GPL 3.0	https://osf.io/3jy46
quadcopter_frame	STL file	GNU GPL 3.0	https://osf.io/tc54f
frame_attachment	STL file	GNU GPL 3.0	https://osf.io/pcets
frame_cover	STL file	GNU GPL 3.0	https://osf.io/bp9aw
frame_bottom_holder	STL file	GNU GPL 3.0	https://osf.io/euht8
esp32_pc	Arduino file	GNU GPL 3.0	https://osf.io/kynp7
esp32_drone	Arduino file	GNU GPL 3.0	https://osf.io/8gq2u
interface	Python file	GNU GPL 3.0	https://osf.io/4q8pb

Below is a short description of the files presented in the table above.

• outer_arc, CAD file for 3D printing part of the yaw gimbal.
• outer_coupling, CAD file for 3D printing part of the yaw gimbal.
• middle_arc, CAD file for 3D printing part of the pitch gimbal.
• middle_coupling1, CAD file for 3D printing part of the pitch gimbal.
• middle_coupling2, CAD file for 3D printing part of the pitch gimbal.
• inner_support1, CAD file for 3D printing part of the roll gimbal.
• inner_support2, CAD file for 3D printing part of the roll gimbal.
• inner_coupling, CAD file for 3D printing part of the roll gimbal.
• quadcopter_frame, CAD file for 3D printing the frame of the quadcopter.
• frame_attachment, CAD file for 3D printing the parts to hold the drone frame to the platform.
• frame_cover, CAD file for 3D printing the cover to hold the ESP32 board.
• frame_bottom_holder, CAD file for 3D printig the bottom part of the quadcopter to hold the battery.
• esp32_pc, Arduino file for the ESP32 board that connects to the PC.
• esp32_drone, Arduino file for the ESP32 board that connects to the drone.
• interface, python file of the interface for controlling the quadcopter and visualize the received data.

## 4 Bill of materials summary

**Table:** 

Component	Number	Cost per unit (USD)	Total cost (USD)	Source of materials
Motor 1804 2480KV CW	2	$6.00	$12.00	https://emaxmodel.com/collections/mt-series/products/emax-multicopter-motor-mt1804-kv2480
Motor 1804 2480KV CCW	2	$6.00	$12.00	https://emaxmodel.com/collections/mt-series/products/emax-multicopter-motor-mt1804-kv2480?variant=35881159065758
Electronic Speed Controller 12A	4	$11.50	$46.00	https://emaxmodel.com/collections/blheli-simon/products/simon-12a-esc_hbs
Flight controller Skyline32	1	$26.99	$26.99	https://emaxmodel.com/products/emax-skyline32-flight-controller-advanced-v1-1
LiPo battery 3S 1300mAH	1	$18.99	$18.99	https://a.co/d/6CSt1qc
XT60 male connector	1	$1.20	$1.20	https://a.co/d/0dgT6iD
5030 Propeller (CW/CCW)	2	$1.01	$2.02	https://a.co/d/1njRckB
ESP32 microcontroller	2	8.99	17.98	https://a.co/d/hKQLi8c
PLA 3D printing filament	1	$22.99	$22.99	https://a.co/d/ewC1KOC
Hex socket screw M3 × 16 mm	32	$0.02	$0.64	https://a.co/d/2cUwAMt
Hex socket screw M3 × 12 mm	14	$0.02	$0.28	https://a.co/d/2cUwAMt
Hex socket screw M5 × 20 mm	2	$0.05	$0.1	https://a.co/d/4pU1kin
Hex socket screw M5 × 25 mm	2	$0.05	$0.1	https://a.co/d/4pU1kin
Hex socket screw M5 × 30 mm	2	$0.05	$0.1	https://a.co/d/4pU1kin
Hex nut M3	46	$0.02	$0.92	https://a.co/d/2cUwAMt
Hex nut M5	18	$0.02	$0.36	https://a.co/d/4pU1kin
Ball bearing 625-2z	6	$0.91	$5.46	https://a.co/d/6FDzlTa
Nylon spacer M3 × 25 mm	4	$0.02	$0.08	https://a.co/d/dC1lYxJ
Jumper wires	1	$2.00	$2.00	https://a.co/d/j3fKaQ2
Steel frame	1	$15.00	$15.00	https://www.homedepot.com/p/Everbilt-1-in-x-6-ft-Plain-Steel-Square-Tube-1009/332735014

The mechanical components (screws, nuts and ball bearings) can be obtained in local hardware stores. The steel frame that holds the platform was custom built, the source link included in the BOM corresponds to the material used for manufacturing. Fig. 4 shows a drawing with dimensions of the fabricated steel frame structure.

**Fig. 2:**
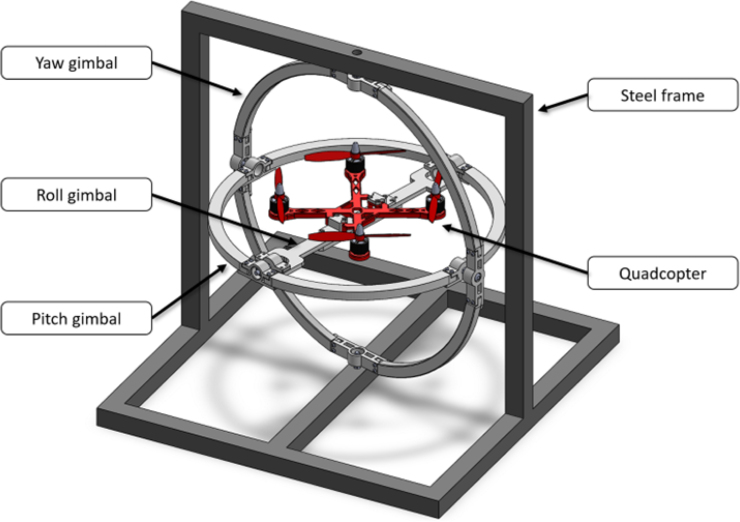
Assembly of the proposed platform for quadcopter control testing.

**Fig. 3:**
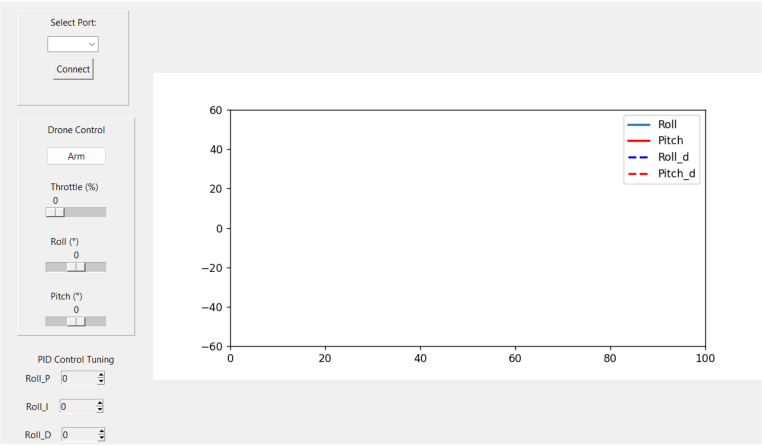
Graphical User Interface for control of quadcopter and visualization of sensors data.

**Fig. 4:**
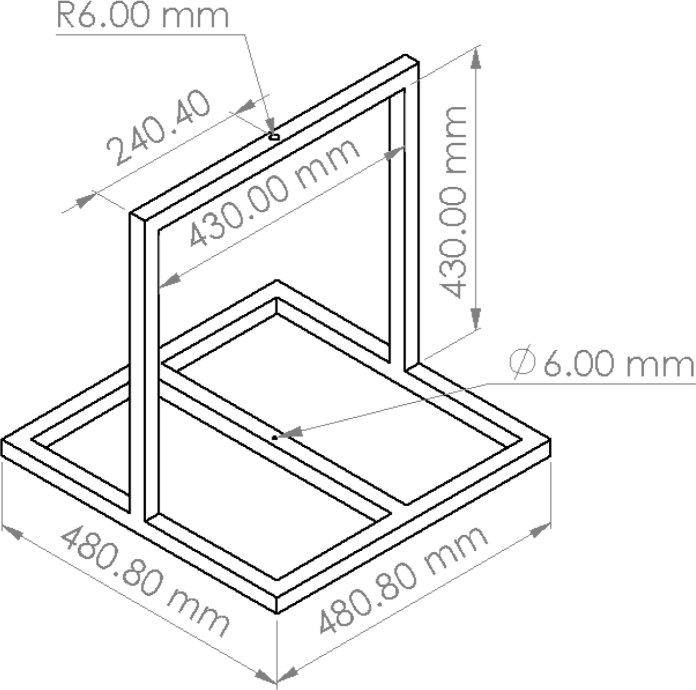
Drawing showing the main dimensions of the fabricated steel structure used as the fixed base for the ARMTOP.

## 5 Build instructions

To manufacture our parts we used a Fused Deposition Modeling 3D printer. Its model is Creality Ender 3 V3 SE. These parts where printed using PLA plastic and the printer was set to print with a later height of 0.2 mm. The top and bottom shells are formed by 4 layers and the walls have 3 loops. All these parts where printed with a 30% infill. All parts were printed with PLA filament because of its rigidity and ease of fabrication. To ensure proper alignment during assembly, the 3D printed components were designed with an acceptable tolerance of ±0.2mm, which provides smooth motion between gimbal joints. This tolerance level accommodates typical variations of fused deposition modeling (FDM) printers as the one used.

The procedure for assembling the quadcopter module is as follows.

**Fig. 5:**
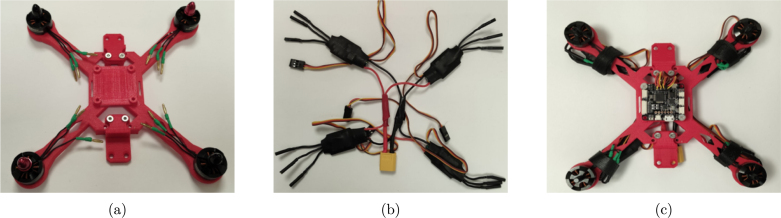
Assembling of drone. (a) Mounting the brushless motors. (b) Soldering all the ESCs to the battery connector. (c) Mounting the flight controller on the center of the drone frame, connecting the motors to the ESCs and the signal of the ESCs to the flight controller.

**Fig. 6:**
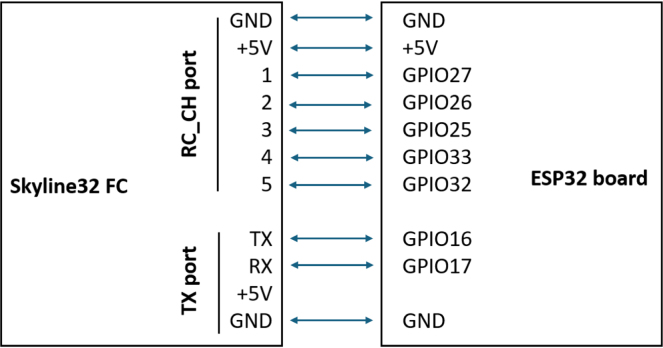
Connection of the Skyline32 flight controller to the ESP32 board.

1. Start by mounting the brushless motors on the 3D printed frame as shown in Fig. 5(a) using the screws supplied with the motors. The front left motor and the back right motor should rotate clockwise, while the front right motor and the back left motor should rotate counterclockwise. It is important to check that the nuts holding the propellers turn in the correct direction so that they do not loosen as they turn.
2. Solder the electronic speed controllers (ESC) to the XT60 connector. Solder the red wires of the ESCs to the positive terminal (＋) of the XT60 connector and the black wires of the ESCs to the negative terminal (−) of the XT60 connector. Make sure to use electrical tape to cover the soldered parts of the wires in order to avoid a short circuit.
3. Connect the ESCs to the motors. The connections can be soldered directly, or bullet connectors can be soldered to the ESCs and motors wires to facilitate the change of the components.
4. Mount the flight controller (FC) on the top of the quadcopter frame. The arrow printed at the bottom of the FC board indicates the front direction of the drone.
5. Connect the signal cable of the ESCs to the flight controller. The yellow wire from the ESC is the signal, the red wire is the power and the black wire is the ground. The back right motor is Motor 1, the front right motor is Motor 2, the back left motor is Motor 3 and the front left motor is Motor 4.
6. Use the cables included with the flight controller and jumper wires to connect it to the ESP32 board according to Fig. 6. The RC_CH port of the flight controller receives the PWM control signals generated with the ESP32. The RT port of the flight controller allows serial communication.
7. Use nylon spacers to place the 3D printed frame_cover on top of the quadcopter frame, securing the ESP32 board.
8. Before installing the propellers, it is important to verify that all motors rotate in the correct direction. This will be done once the quadcopter is mounted on the platform.

The following are the required steps for the assembly process of the Mrotor Dock.

**Fig. 7:**
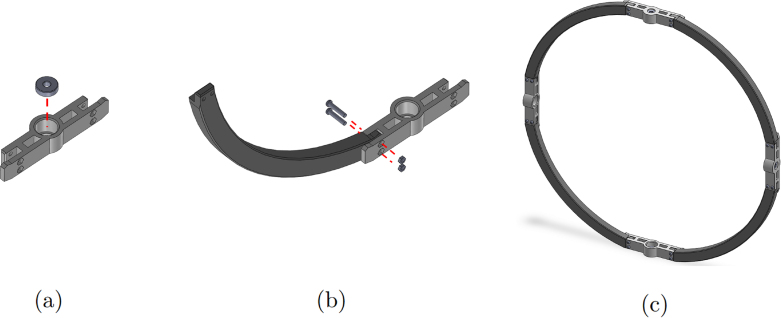
Assembly of the yaw gimbal. (a) insertion of the ball bearing. (b) attachment of the outer_arc and outer_coupling parts. (c) yaw gimbal ring assembled.

**Fig. 8:**
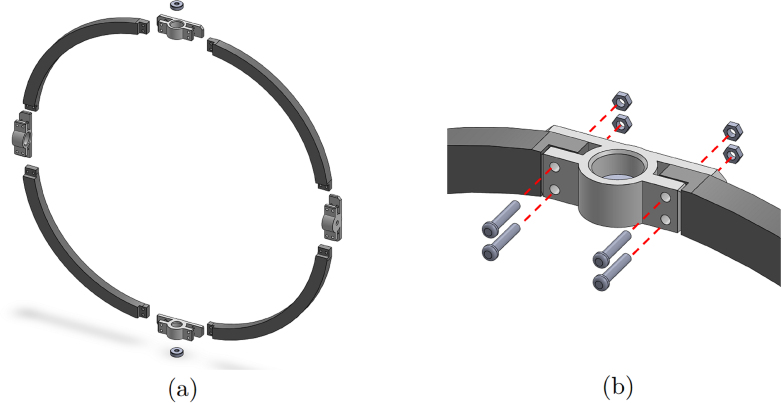
Assembly of the pitch gimbal. (a) Assembling of the ring showing the position of the ball bearings. (b) Detailed view of the assembly, using M3 × 16 mm screws and M3 nuts.

**Fig. 9:**
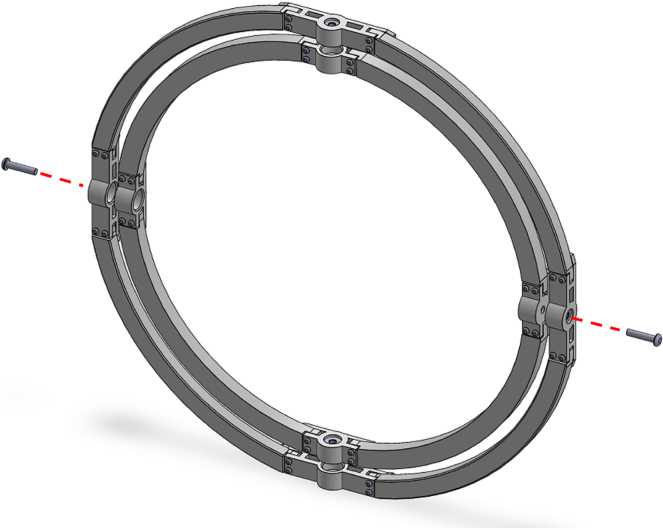
Connection of yaw and pitch gimbal.

**Fig. 10:**
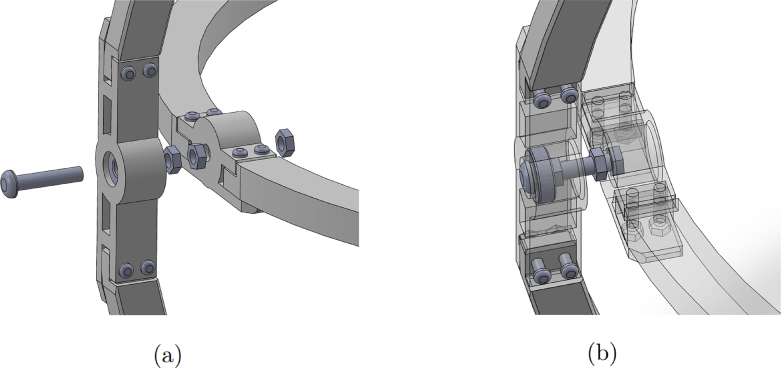
Detailed view of yaw and pitch gimbal connection. (a) Insertion of M5 × 25 mm screws and M5 nuts to connect yaw and pitch gimbals. (b) Transparent view to show the position of the screws and nuts in the assembly.

**Fig. 11:**
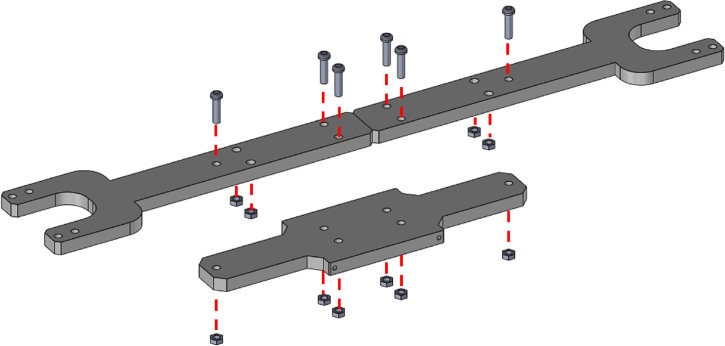
Assembly of roll gimbal.

**Fig. 12:**
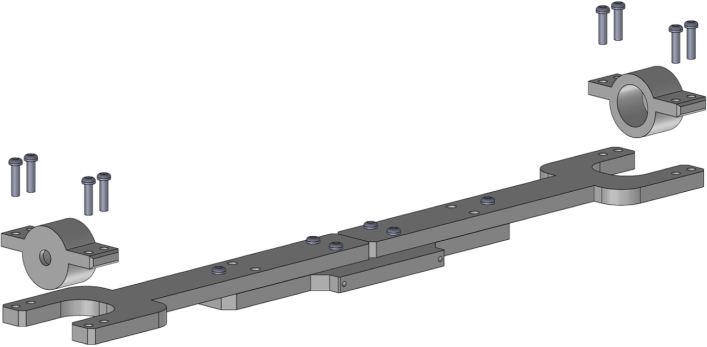
Assembly of roll gimbal.

**Fig. 13:**
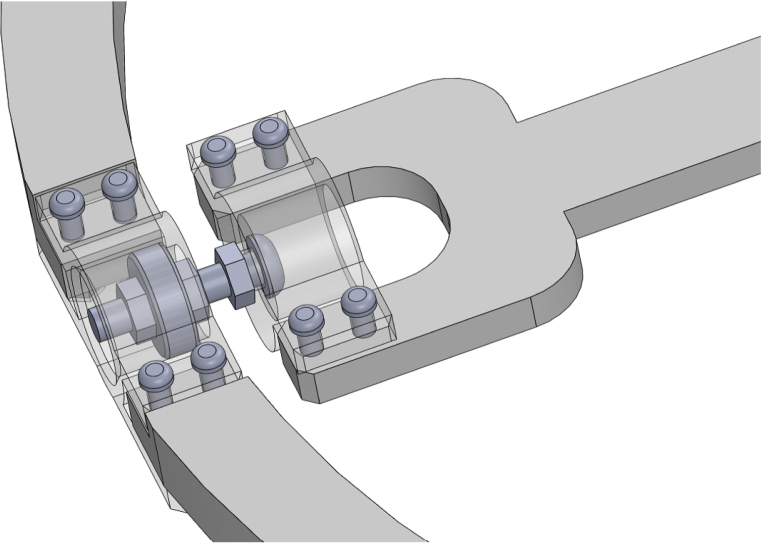
Detailed view of roll and pitch gimbal connection.

**Fig. 14:**
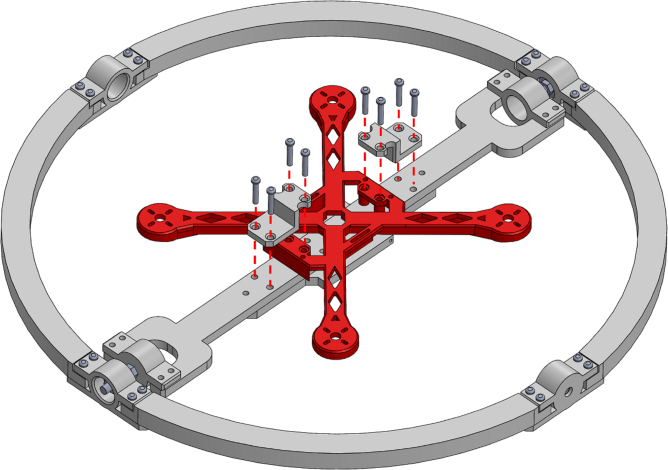
Quadcopter frame attachment.

1. Start by inserting the ball bearings into the “outer_coupling” parts (Fig. 7(a)).
2. Attach the four “outer_arc” parts to the “outer_coupling” parts using M3 × 16 mm screws and nuts (Fig. 7(b)), to form the ring of the yaw gimbal as shown in Fig. 7(c).
3. The assembly of the pitch gimbal is similar, inserting two ball bearings into the parts named “middle_coupling1” in the positions shown in Fig. 8(a). Assemble the ring with the four “middle_arc”, the two “middle_coupling1” and two “middle_coupling2” parts, using M3 × 16 mm screws and M3 nuts as shown in Fig. 8(b).
4. The connection between the yaw and pitch gimbal uses two M5 × 25 mm screws according to Fig. 9. Three M5 nuts are used on each screw to hold the two rings with equal spacing. A detailed view of this connection can be seen in Fig. 10.
5. The roll gimbal is made up of 3 parts. Start by placing M3 nuts in the hex slots of the parts and use M3 × 12 mm screws to attach the parts together (Fig. 11).
6. Attach the inner_coupling part to the roll gimbal using M3 × 12 mm screws and M3 nuts as shown in Fig. 12.
7. Fig. 13 shows a detailed view of the connection between roll gimbal and pitch gimbal, using two M5 × 20 mm screws and three M5 nuts on each screw to keep the spacing between the two parts.
8. The quadcopter frame designed can be mounted in the roll gimbal using the frame_attachments and M3 × 16 mm screws (Fig. 14).
9. Finally, the assembled set of gimbals is mounted in a sturdy frame using M5 screws in the free ball bearings of the yaw gimbal (Fig. 2).

.

## 6 Operation instructions

### 6.1 Telemetry and command module

**Fig. 15:**
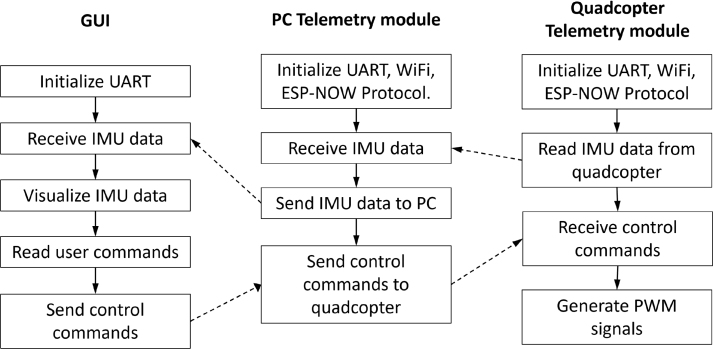
Software operation flowchart.

The firmware for the Telemetry and Command Module (TCM) was developed and compiled using the Arduino IDE [18] version 2.3.2 with the ESP32 board support package version 1.0.6. Two libraries were employed to implement wireless communication. (a) esp_now.h, for low-latency, peer-to-peer communication between ESP32 modules. (b) WiFi.h, to configure device roles and manage network initialization. Every ESP32 board has a unique Media Access Control Address that identifies the device on a network. A sketch can be used to find the MAC address of the boards to use [19]. After loading the file with the Arduino IDE and uploading it to the ESP32 board, open the Serial Monitor of the IDE at a baud rate of 115200 and restart the board. The MAC Address should be printed in the Serial Monitor. In the “esp32_dron.ino” and “esp32_pc.ino” files, a broadcast address is used to transmit data to all devices in the network. This address is represented as FF:FF:FF:FF:FF:FF in hexadecimal. This address can be changed to the unique address of each device. In the “esp32_pc.ino” put the address of the board that will be connected to the drone. In the “esp32_dron.ino” put the address of the board that will be connected to the PC. Fig. 15 shows the flowchart between the GUI and the TCM modules.

### 6.2 Graphical user interface

The Graphical User Interface (GUI) for real-time control, data visualization and logging was developed in Python 3.9 using open-source libraries to ensure cross-platform compatibility and ease of customization. The GUI was tested on Windows 10 operating system. Six packages are required for proper execution. (a) tkinter, for the graphical interface elements. (b) pyserial, for establishing communication with the ESP32 telemetry module via USB. (c) matplotlib, for real time plotting. (d) threading, for concurrent data acquisition and visualization. (e) csv, for structured data export. (f) datetime, for timestamping the recorded measurements.

Upon execution of the Python code, the GUI shows in the left side the controls and in the right side the graph of the sensor data (see Fig. 3). The top left side has a drop-down list to select the serial port of the ESP32 board connected to the PC, and a button to start the connection. Below this, the drone control section has a button to arm the drone and sliders to increase the throttle and select the desired roll and pitch angles. In bottom left side, the PID control tuning section allows to modify the PID gains of the roll control system. The right side of the GUI displays the roll and pitch angle data received from the drone and also the desired roll and pitch angles.

### 6.3 Drone operation

The quadcopter module integrated into the testing platform employs a Skyline32 flight controller board, which is compatible with the Cleanflight firmware environment. The configuration process follows standard procedures described in the official Skyline32 User Manual (available at http://yinyanmodel.com/EN/DownView.asp?ID=58). Firmware setup and tuning were performed using the Cleanflight Configurator version 2.6.0, an open-source graphical interface available at https://github.com/cleanflight/cleanflight-configurator/releases. For initial connection and configuration through USB, users must install the CP210x USB-to-UART Bridge VCP drivers provided by Silicon Labs, available at https://www.silabs.com/developers/usb-to-uart-bridge-vcp-drivers.

Connect the battery to power on the drone. The led lights in the flight controller board will start blinking while calibration of onboard sensors occur. It is important to keep the drone still and level during this process. Once the green led light stops blinking, the drone is ready for use. Before installing the propellers, use the python interface to arm the motors and increase the throttle to verify that each motor rotates in the correct direction. Motors 1 and 4 should rotate clockwise, while motors 2 and 3 should rotate counter clockwise. In case a motor rotate in the wrong direction, interchanging a pair of wires between the motor and the ESC will change the rotation direction. Once all the motors rotate in the correct direction, the propellers can be installed making sure the correct propeller is used according to the rotate direction of each motor.

### 6.4 Calibration procedure

Proper calibration of the onboard sensors is essential to ensure accurate attitude estimation and stable flight control. The Skyline32 flight controller integrates a three-axis accelerometer and gyroscope within its inertial measurement unit (IMU). The following calibration steps were performed prior to experimental testing using the Cleanflight Configurator (v2.6.0) environment and the CLFL 1.10.0 firmware.

Accelerometer calibration

The accelerometer calibration was conducted by placing the platform on a perfectly level and stable surface, ensuring that no motion occurred during the process. In the Cleanflight Configurator, the “Calibrate Accelerometer” command was executed under the Setup tab. This operation automatically determined the zero-offset values for each axis and stored them in the flight controller’s non-volatile memory. The calibration ensures that the pitch and roll angles are correctly measured when the platform is in a horizontal position, minimizing bias due to sensor drift or mounting imperfections.

Gyroscope calibration

Gyroscope calibration was automatically performed during each power cycle after the flight controller initialization. To ensure optimal results, the platform was kept completely motionless for approximately 10 s while the status LED indicated the calibration sequence. This step compensates for the sensor’s zero-rate output (bias) on all three rotational axes, improving angular rate precision during subsequent experiments.

After both procedures, the IMU data was verified through GUI to confirm stable readings near zero for all static axes. These calibration steps are critical for achieving repeatable and reliable measurements in the test platform. Detailed instructions for performing both procedures are also available in the official Skyline32 Flight Controller Manual.

Calibration verification

**Fig. 16:**
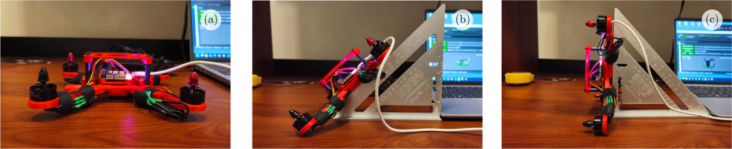
Verification of the inertial sensor calibration. The drone was positioned at inclination angles of (a) 0°, (b) 45°, and (c) 90°using a right-angle square as a mechanical reference. These orientations were used to confirm that the angles displayed in the graphical interface closely matched the physical inclinations of the platform after calibration.

Once calibration was completed, verification was performed by positioning the drone at three different inclination angles as illustrated in Fig. 16. Specifically the drone is positioned at 0° Fig. 16(a), 45° Fig. 16(b) and 90° Fig. 16(c), using a right angle square as mechanical reference. The angles displayed in the GUI were compared with the physical inclinations, which confirmed that the measured values closely matched the expected orientations. This procedure validated the accuracy of the calibration process and the correct response of the inertial sensors.

**Fig. 17:**
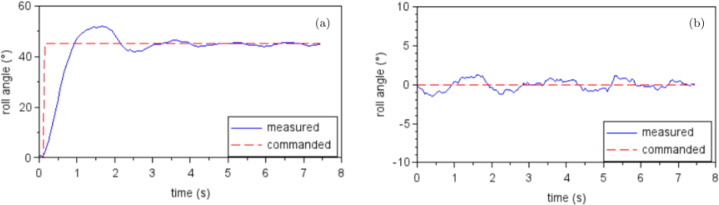
Validation test showing the drone roll angle at (a) 45°and (b) 0°. This positions were used to compare the measured angular response with the expected reference value, with average steady-state errors of 0.105°and 0.060°respectively. The average time to reach steady state was 2.95 s.

Once the calibration was completed and the drone was mounted on the platform, the performance of the system was validated under controlled laboratory conditions (indoors, no wind, constant illumination) to minimize external disturbances. During the tests, a sequence of flight commands was issued through the control interface to move the drone to specific orientations of 0° Fig. 17(a) and 45° Fig. 17(b), allowing comparison between the commanded and measured angular positions as shown in Fig. 17.

### 6.5 Common issues and troubleshooting

During testing, three main issues were identified as potential sources of malfunction.

• Serial connection errors, communication failures between the ESP32 telemetry module and the GUI may occur if the incorrect serial port is selected. Users should verify the assigned COM port in the operating system and confirm that the correct one is selected within the GUI interface before establishing communication.
• Telemetry display delay, delays or interruptions in the real-time visualization of data may result from limited system memory or concurrent background processes on the host computer. Closing unnecessary applications or increasing available RAM typically resolves this issue.
• Reduced system performance, slow response or irregular behavior of the platform can be caused by insufficient battery charge. It is recommended to check battery voltage before each operation and ensure full charging to maintain stable flight control and sensor response.

Regular maintenance of the mechanical components is essential to preserve the platform’s precision and minimize wear. The following preventive actions are recommended.

• Lubrication, apply a small amount of lubricant oil to the gimbal bearings when friction is detected.
• Fastener adjustment, periodically verify that all screws and nuts remain firmly tightened, especially at bearing joints and structural couplings, to prevent undesired mechanical play.
• Battery care, recharge the LiPo battery after each session and avoid deep discharge below 3.2 V per cell to extend its lifespan and ensure consistent power delivery.

## 7 Validation and characterization

This section focuses on validating the performance of ARMTOP through a representative use case, tuning the PID parameters. Additionally, the moments of inertia of our platform are calculated and presented to aid in describing its rotational dynamics.

### 7.1 Use case: Tuning PID parameters

**Fig. 18:**
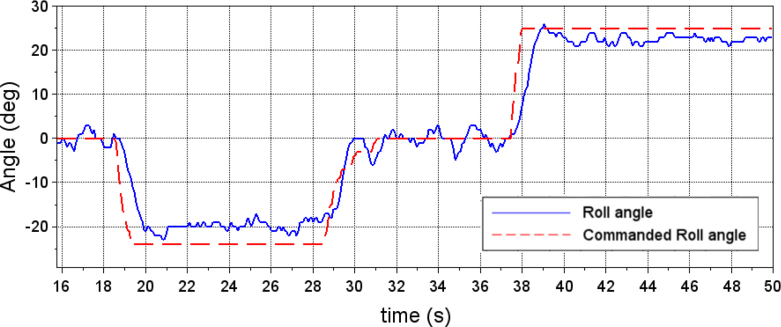
Quadcopter response to commanded roll angle before PID tuning.

To demonstrate the operation of the hardware, an experimental test is performed to tune the PID gains of the roll system. In a first instance, incorrect PID gains are entered into the system and a desired roll angle is commanded. In Fig. 18, the data stored by the PC interface was used to graph the system response to this experiment. The red dotted line represents the desired angle, entered into the drone control section of the PC interface. The blue line shows the quadcopter’s response to the desired input. In this case, the PID gains entered for the control of the roll angle where kP=5, kI=5 and kD=5. The graph shows oscillations in the quadrotor response when the desired angle is 0°(hover state)and a noticeable error in steady state for the desired angles of −25°and 25°.

**Fig. 19:**
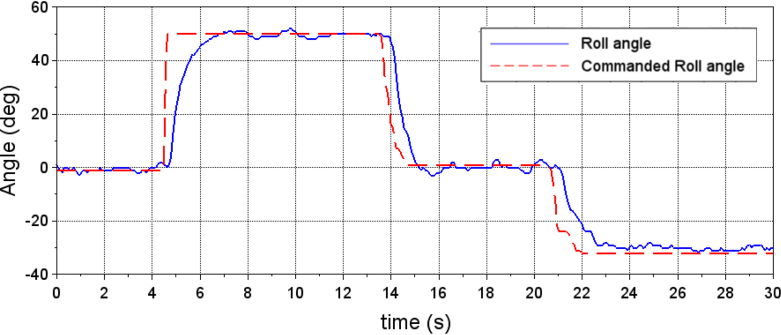
Quadcopter response to commanded roll angle after PID tuning.

Using the PID control tuning section of the PC interface, it is possible to modify the PID gains of the roll angle controller. Using a manual tuning approach, the final gains introduced were kP=22, kI=20 and kD=30. Fig. 19 shows the response of the quadcopter to the desired roll angle. In this case the system response is smoother and the steady-state error is smaller.

Without the use of the ARMTOP platform or prior knowledge of the quadcopter’s response, performing this controller tuning can be dangerous due to the quadcopter’s movements.

### 7.2 Rotational dynamics of the platform

The moments of inertia of a vehicle quantify important information regarding the weight distribution of the vehicle and its ability to rotate around various axes. They may also be necessary to adequately simulate the motion of the vehicle. If a multi-rotor drone is being tested in our platform, the moments of inertia may be obtained from controlled flight tests in the platform. However, these moments of inertia include both the moments of inertia of the drone and the moments of inertia of the gimbals that hold the drone. To obtain the moments of inertia of just the multi-rotor drone, which are relevant to describe free flight without the platform, we need to subtract the moments of inertia of the gimbals from the moments of inertia extracted from flight in the platform. Therefore, as part of the characterization of our device, we calculate the moments of inertia of the gimbals that hold the multi-rotor drone.

**Fig. 20:**
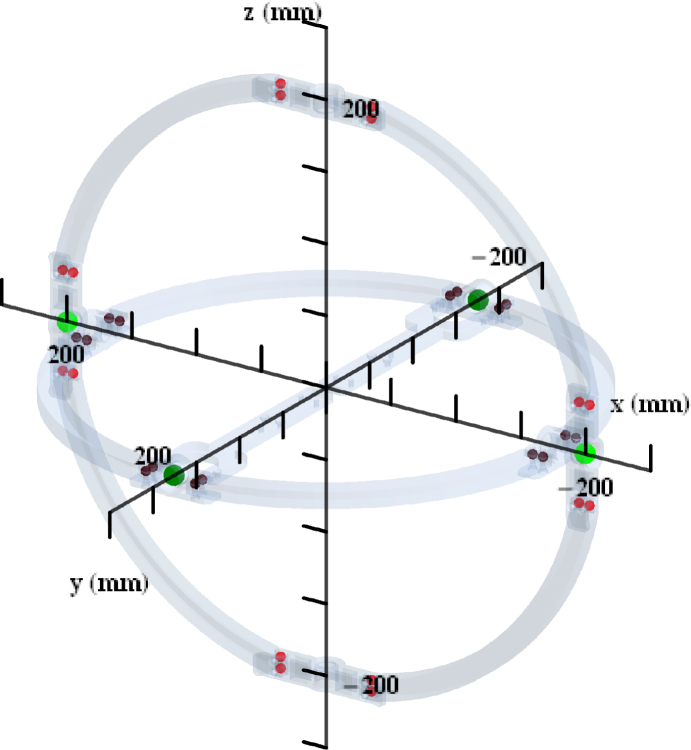
Gimbals, axes, and positions of bolt-nut pairs and bearings are shown. Bolt-nut pairs appear as red dots, bearings as green dots. Light-colored dots contribute to the yaw gimbal inertia matrix, $I_{\text{g,yaw}}$, while dark-colored dots contribute to the pitch gimbal $I_{\text{g,pitch}}$.

The gimbals in our platform rotate as the drone being tested rolls, pitches or yaws. Therefore, the matrix of the moments of inertia for such objects may be useful when characterizing the moments of inertia of a multi-rotor drone that is being tested in this platform. These moments of inertia are calculated for rotations around the geometric center of the gimbals as displayed in Fig. 20. We set the x axis as the horizontal right direction, the y axis as the horizontal direction going inside the page, and the z axis as the vertical upwards direction.

The yaw, pitch, and roll frames respectively have the following moments of inertia matrices

(1) $$I_{\text{g,yaw}}=\left(\begin{array}{ccc} 22\;400 & 0 & 0\\ 0 & 48\;000 & 0\\ 0 & 0 & 25\;600 \end{array}\right)\;I_{\text{g,pitch}}=\left(\begin{array}{ccc} 18\;300 & 0 & 0\\ 0 & 15\;800 & 0\\ 0 & 0 & 34\;000 \end{array}\right)I_{\text{g,roll}}=\left(\begin{array}{ccc} 1700 & 0 & 0\\ 0 & 20.1 & 0\\ 0 & 0 & 1710 \end{array}\right),$$

in units of $\mathrm{gr}\;{\mathrm{cm}}^{2}$. These matrices where calculated assuming a homogeneous material with 30% the density of PLA plastic, $\rho_{\text{PLA}}=1.24\;\mathrm{gr}/{\mathrm{cm}}^{3}$. This is because these objects where printed in PLA plastic with 30% filling. Bolts and nuts contribute to the moments of inertia. These contributions are included in the matrices above as point masses with 1.23gr each. Two bearings connect the yaw and pitch gimbals, and they rotate with the yaw gimbal. Their contribution to the moments of inertia of the yaw gimbal is also included in $I_{\text{g,yaw}}$. These two bearings are assumed as point masses with mass 4gr, each. The two bearings that are connected the steel frame and the yaw gimbal do not rotate with the gimbal and are therefore no included in its moments of inertia. Two bearings connect the pitch and roll gimbals, and they rotate with the pitch gimbal. Their contribution is included in $I_{\text{g,pitch}}$ also as point masses with mass 4gr. The position of these bolts, nuts and bearings is displayed in Fig. 20.

**Fig. 21:**
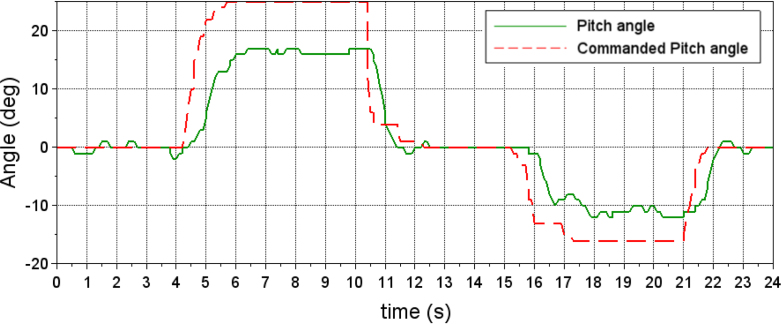
Quadcopter response to commanded pitch angle after PID tuning.

Since the quadcopter is symmetrical, the gains used for roll angle control are usually very close or equal to the gains for pitch angle control. In the case of the ARMTOP platform, one limitation is that the inertia of the pitch-angle mechanism is greater than the inertia of the roll-angle mechanism. This causes the gains used for the roll-angle PID control to not have the same response when used for the pitch-angle control. As shown in Fig. 21, when adjusted gains for roll-angle control are used, the quadrotor response for a desired pitch angle exhibits a considerable offset in steady state.

## CRediT authorship contribution statement

**Mario Aguilera-Ruiz:** Writing – original draft, Software, Methodology, Investigation, Formal analysis, Data curation, Conceptualization. **Alejandro Galaviz-Mosqueda:** Writing – review & editing, Supervision, Resources, Project administration, Methodology, Investigation, Funding acquisition, Conceptualization. **Benjamín Jaramillo-Ávila:** Writing – review & editing, Visualization, Validation, Methodology, Formal analysis. **Salvador Villarreal-Reyes:** Writing – review & editing, Investigation, Conceptualization, Project administration.

## Declaration of competing interest

The authors declare that they have no known competing financial interests or personal relationships that could have appeared to influence the work reported in this paper.
